# Paradigm Shift in Sensorimotor Control Research and Brain Machine Interface Control: The Influence of Context on Sensorimotor Representations

**DOI:** 10.3389/fnins.2018.00579

**Published:** 2018-09-10

**Authors:** Yao Zhao, John P. Hessburg, Jaganth Nivas Asok Kumar, Joseph T. Francis

**Affiliations:** ^1^Joint Program in Biomedical Engineering, Polytechnic Institute of NYU and SUNY Downstate, Brooklyn, NY, United States; ^2^Department of Biomedical Engineering, Cullen College of Engineering, University of Houston, Houston, TX, United States; ^3^Department of Physiology and Pharmacology, Robert F Furchgott Center for Neural and Behavioral Science, State University of New York Downstate Medical School, Brooklyn, NY, United States

**Keywords:** reinforcement learning, brain machine interface (BMI), motor cortex, somatosensory cortex, dopamine, sensorimotor control

## Abstract

Neural activity in the primary motor cortex (M1) is known to correlate with movement related variables including kinematics and dynamics. Our recent work, which we believe is part of a paradigm shift in sensorimotor research, has shown that in addition to these movement related variables, activity in M1 and the primary somatosensory cortex (S1) are also modulated by context, such as value, during both active movement and movement observation. Here we expand on the investigation of reward modulation in M1, showing that reward level changes the neural tuning function of M1 units to both kinematic as well as dynamic related variables. In addition, we show that this reward-modulated activity is present during brain machine interface (BMI) control. We suggest that by taking into account these context dependencies of M1 modulation, we can produce more robust BMIs. Toward this goal, we demonstrate that we can classify reward expectation from M1 on a movement-by-movement basis under BMI control and use this to gate multiple linear BMI decoders toward improved offline performance. These findings demonstrate that it is possible and meaningful to design a more accurate BMI decoder that takes reward and context into consideration. Our next step in this development will be to incorporate this gating system, or a continuous variant of it, into online BMI performance.

## Introduction

Primary motor cortical (M1) activity encodes movement related kinematics and dynamics (Georgopoulos et al., 1982, 1992), and is often modeled as a linear relationship between neuronal firing and desired movement toward the development of brain-machine interfaces (BMIs) (Chhatbar and Francis, 2013). BMIs allow subjects to control physical or virtual systems including robotic arms and computer cursors using neural signals (Serruya et al., 2002; Taylor et al., 2002; Velliste et al., 2008; Li et al., 2011). BMIs have been used to restore reaching and grasping for paralyzed patients with some success (Hochberg et al., 2012; Collinger et al., 2013; Bouton et al., 2016; Ajiboye et al., 2017), and making such systems more robust and easier to control is of great importance. A critical component of BMIs are the motor cortex tuning models, which describe the relationship between neural firing rates and kinematics, such as hand/endpoint velocity (Taylor et al., 2002), or dynamics, such as force and torques (Carmena et al., 2003; Chhatbar and Francis, 2010, 2013; Suminski et al., 2011).

Recently, our lab and others have shown that context can modulate neural activity in M1 (Chhatbar and Francis, 2013; Marsh et al., 2015; Ramkumar et al., 2016; Downey et al., 2017; Ramakrishnan et al., 2017). It has become clear that a reward signal exists in the primary motor cortex as well as the primary somatosensory cortex (McNiel et al., 2016a,b). Additionally, context such as reward level can affect direction tuning curves for M1 units (Ramakrishnan et al., 2017). We hypothesized (Marsh et al., 2015) that this reward signal originates in midbrain dopaminergic areas such as the ventral tegmentum (VTA) and substantia nigra pars compacta (SNc), as there are dopaminergic receptors as well as terminals from these dopaminergic centers in M1 (Richfield et al., 1989). Dopamine is necessary for LTP in M1 (Molina-Luna et al., 2009), and has been shown to have a “charging” effect on neural activity, possibly acting as a motivational signal (Hollerman and Schultz, 1998; Schultz, 2000; Hamid et al., 2016). Although the aforementioned studies showed that M1 neurons multiplex the representation of reward and motor activity, it has yet to be concretely characterized whether such reward modulation could be used to design a more robust and accurate BMI decoder. In the current study, we demonstrate for the first time to our knowledge that reward modulates neural activity related to dynamic variables, such as grip force, and to BMI controlled kinematic variables, such as velocity.

The current work has two main goals. First, to show that significant differences exist in both directional and force tuning models of M1 units between rewarding and non-rewarding trials, and second, the reward level (Tarigoppula et al., 2018), that is the value of a given movement, can be used as additional information to improve BMI decoding accuracy in an offline, open-loop system where rewarding and non-rewarding trials are classified and decoded separately.

## Methods

### Surgery

Two non-human primates (NHPs), one male rhesus macaque (monkey S) and one female bonnet macaque (monkey P), were implanted with chronic 96-channel platinum microelectrode arrays (Utah array, 10 × 10 array separated by 400 μm, 1.5 mm electrode length, ICS-96 connectors, Blackrock Microsystems). The hand and arm region of M1 contralateral to their dominant hand was implanted with the same technique as our previous work (Chhatbar et al., 2010; Marsh et al., 2015). All surgical procedures were conducted in compliance with guidelines set forth by the National Institutes of Health Guide for the Care and Use of Laboratory Animals and were approved by the State University of New York Downstate Institutional Animal Care and Use Committee. Briefly, animal preparation and the induction and maintenance of anesthesia were conducted by members of the State University of New York Downstate Division of Comparative Medicine veterinary staff. Aseptic conditions were maintained throughout the surgery. Ketamine was used to induce anesthesia, and isofluorane and fentanyl were used for maintenance. Dexamethasone was administered to prevent inflammation during the procedure, and diuretics including mannitol and furosemide were available to further reduce cerebral swelling if needed. Both subjects were observed hourly for the first 12 h after implantation, and provided with a 7-day course of antibiotics (baytril and bicilin) and analgesics (buprenorphine and rimadyl).

### Extracellular unit recordings

After a 2–3 week recovery period, spiking activity was recorded with a multichannel acquisition processor system (MAP, Plexon Inc.) while the subjects performed the experimental task. Neural signals were amplified and bandpass filtered between 170 Hz and 8 kHz to isolate single and multi-unit activity and sampled at 40 kHz, and each channel manually thresholded to detect single units. Single and multi-units were sorted based on their waveforms using principal component (PC)-based methods in Sort-Client software (Plexon Inc.).

### Behavioral task

**Figure 1:**
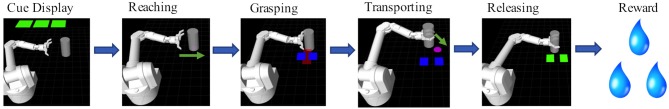
Behavioral Task: The behavioral task was composed of 6 scenes. First, during the cue display scene the animal was cued via the number of green squares to the amount of reward it would receive if it completed a trial successfully. Each green square indicated 0.5 s worth of liquid reward. Zero green squares indicated a non-rewarding trial. Trials could either be under manual control or BMI control. In manual control the NHP squeezed a physical manipulandum, with the amount of force, represented by a red rectangle, having to be held within the blue target rectangles in order for the trial to be successful. In BMI control mode, the NHP controlled the reaching trajectory of the arm toward the object (see Methods section).

Monkeys S and P were trained to perform a reach-grasp-transport-release task, depicted in Figure 1. In this task, the subjects controlled certain aspects of a simulated anthropomorphic robotic arm (Barrett WAM) in order to manipulate a virtual target cylinder. Each trial consisted of 6 stages: cue display, reaching, grasping, transporting, releasing, and reward delivery. At the start of a trial, cues were displayed (green squares) to indicate the level of juice reward the animal would receive upon successful completion of the task. The number of green squares (0–3) corresponded to the number of 0.5 s juice delivery periods delivered at the end of that trial. If no green square was displayed, then no reward was delivered upon successful completion of the trial. In an unsuccessful trial, no reward was delivered and the trial was repeated at the same reward level until completed successfully, motivating subjects to complete the zero reward level trials successfully. Two NHPs conducted two sessions each of a manual grip force control version of the task as well as two sessions each of a BMI version of the task.

For the manual task, the virtual arm reached the target cylinder automatically. The animal then controlled the grasping motion of the hand by manually squeezing a force transducer with its dominant hand. The amount of force applied was represented in the virtual environment by a red rectangle that increased in width proportional to the force output. The subject had to maintain a level of force indicated by a pair of blue force target rectangles (Figure 1). The robotic arm then automatically moved the cylinder to a target location while the animal maintained the target grip force. The animal then released the gripper, which resulted in a successful trial if completed at the proper time, and the cylinder was placed at the target location.

For the BMI task, after reward cue presentation the subject controlled the virtual robotic arm's movement from the starting position to the target cylinder using M1 activity. During the reaching stage of the task, the cylinder was always located horizontally to the right from the starting position of the virtual hand. When within a threshold distance, the cylinder was grasped automatically. The animal then needed to move the cylinder to the target position using BMI control. The target location was determined pseudorandomly within the confines of the task plane. If the animal brought the cylinder to the target position, the trial was considered successful. The hand then automatically released the grasp on the cylinder, and the arm reset to the starting position.

This study was designed to investigate the effect of varying the level of reward on neural encoding, so there were two versions for both BMI and manual tasks, differing in the reward levels offered during the recording session. The amount of juice reward delivered was determined by the amount of time the juice reward straw (electronically controlled by a solenoid) was kept open. The solenoid was opened for 0.5 s for every successive level of reward (approximately 1 ml juice). For the first recording session, the reward levels were 0 (non-rewarding) or 1 (0.5 s of reward delivery). For the second session, the reward levels were 0 and 3 (1.5 s of reward delivery). Only successful trials were considered for further analysis for both BMI and the manual tasks.

During BMI trials, subjects controlled the virtual arm movement during the reaching and transporting stages using a ReFIT Kalman filter (Gilja et al., 2012) BMI decoder, which used binned firing rates (100 ms bins) to predict the animal's command for the virtual arm's velocities. The Kalman filter is a linear dynamical system, which has a state model and observation model.

At time *t*, the state model is:

$$\begin{array}{l} x_{t}=Dx_{t-1}+q_{t},\;q_{t}\sim N\left(0,Q\right), \end{array}$$

and the observation model is:

$$\begin{array}{l} r_{t}=Ex_{t}+w_{t},\;w_{t}\sim N\left(0,W\right), \end{array}$$

where **bold** type represents vectors. ***x***_*t*_ is the state vector representing positions and velocities at time bin *t*, **r**_*t*_ is the observation variable representing binned firing rates at time bin *t*, and ***q***_**t**_ and ***w***_**t**_ are Gaussian noise. Given *D, Q, E, and W*, the Kalman filter can provide the best estimation for the current state ***x***_*t*_ based on the previous state ***x***_*t*−1_ and the current observation ***r***_*t*_. The ReFIT Kalman filter allows one to retrain and improve parameters *D, Q, E*, and *W* using intended velocity data from previous BMI control sessions (Gilja et al., 2012). The bin size used here was 100 ms. The Re-fit Kalman decoder was retrained every block (8000 time bins, 800 seconds).

**Figure 2:**
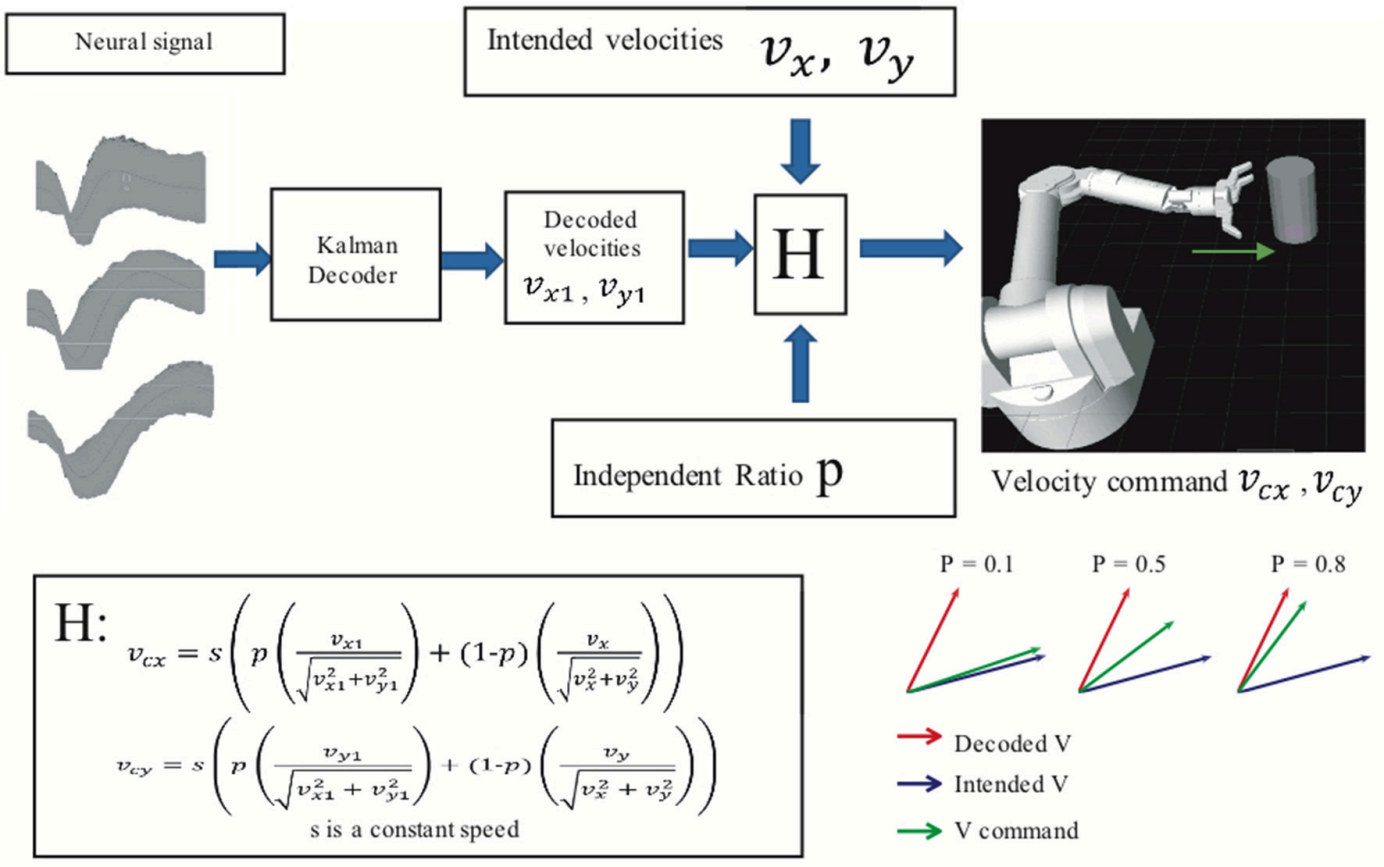
Assisted BMI control. Velocity command is the linear combination of predicted velocities (output from the ReFIT Kalman decoder) and intended velocities. The velocity of the virtual hand is a linear combination (H) of the ReFIT Kalman decoder output and the intended velocity. The higher the value of *p*, the closer the velocity command is to the decoder output.

An assistive controller (Figure 2) was used to modulate the difficulty of the BMI task. In assistive control, the velocity commands *v*_*cx*_ and *v*_*cy*_ that controlled the virtual arm's movement in the x and y dimensions were a linear combination (H in Figure 2) of BMI decoded velocities *v*_*x*1_ and *v*_*y*1_ and “intended” velocities *v*_*x*_ and *v*_*y*_ given by

$$\begin{array}{l} v_{cx}=s\left(p\left(\frac{v_{x1}}{\sqrt{v_{x1}^{2}+v_{y1}^{2}}}\right)+\left(1-p\right)\left(\frac{v_{x}}{\sqrt{v_{x}^{2}+v_{y}^{2}}}\right)\right),\text{and}\\ v_{cy}=s\left(p\left(\frac{v_{y1}}{\sqrt{v_{x1}^{2}+v_{y1}^{2}}}\right)+\left(1-p\right)\left(\frac{v_{y}}{\sqrt{v_{x}^{2}+v_{y}^{2}}}\right)\right), \end{array}$$

where *s* was a constant speed, manually set at 40 *cm*/*s*. Animals reached the cylinder in approximately 0.5 s at that speed.

We define intended velocities as velocity in the direction of the target location, but with the speed of the decoded velocities. Thus, the difficulty of the task could be changed by adding more or less of the intended velocity by adjusting the independence ratio P. A higher value of *P* indicates that the velocity command relied more on the BMI decoder output than the intended velocities. All BMI data used here were recorded in sessions with an independence ratio greater than 0.8.

### Off-line data analysis

#### Tuning curve analysis

Linear regression was performed to fit neural encoding models for both the manual task and the BMI task. For the BMI task, during the reaching and transporting scenes, the linear intended velocity-encoding model was given by:

(1) $$\begin{array}{r} r_{it}=A_{1i}v_{xt}+B_{1i}v_{yt}+C_{i} \end{array}$$

where *r*_*it*_ was unit *i*'s binned firing rate at time bin *t* (100 ms bins). *v*_*xt*_ and *v*_*yt*_ were the BMI controlled virtual hand's intended velocities at time bin *t* in the x and y directions, respectively. The intended velocity at time bin *t* had the same speed as the BMI controlled velocity at time bin *t*, but the direction was toward the target (Gilja et al., 2012). If considering movement direction rather than velocity, we have:

(2) $$\begin{array}{r} r_{it}=A_{2i}cos\left(\theta_{t}-\theta_{pi}\right)+C_{i} \end{array}$$

where θ_*t*_ indicates the intended movement direction, which was the direction toward the target at time bin *t*. θ_*pi*_ was the preferred direction of the *i*th unit, defined as the direction of movement that evoked the maximum firing rate in that unit. Equation (2) is referred to as the intended directional tuning curve equation and can be used for BMI decoding (Moran and Schwartz, 1999). In this study, the virtual hand was always moving at the same speed. Let:

$$\begin{array}{lll} A_{2i} & = & \sqrt{\left(A_{1i}^{2}+B_{1i}^{2}\right)\left(v_{xt}^{2}+v_{yt}^{2}\right)}\\ tan\left(\theta_{pi}\right) & = & \frac{B_{1i}}{A_{1i}},\text{and}\\ tan\left(\theta_{t}\right) & = & \frac{v_{yt}}{v_{xt}}, \end{array}$$

therefore Equation (1) is equivalent to Equation (2).

Intended velocities and directions were used instead of real velocities and directions for fitting Equations (1) and (2) because it has been previously shown that a ReFIT Kalman decoder that makes use of intended kinematics information can correct model parameters and have better BMI performance (Gilja et al., 2012).

Considering rewarding and non-rewarding trials separately, the following equations were written:

(2.1.1) $$\begin{array}{r} r_{it}=A_{1i}^{r}v_{xt}^{r}+B_{1i}^{r}v_{yt}^{r}+C_{1i}^{r} \end{array}$$

(2.1.2) $$\begin{array}{r} r_{it}=A_{2i}^{r}cos\left(\theta_{t}-\theta_{pi}^{r}\right)+C_{2i}^{r} \end{array}$$

(2.2.1) $$\begin{array}{r} r_{it}=A_{1i}^{nr}v_{xt}^{nr}+B_{1i}^{nr}v_{yt}^{nr}+C_{1i}^{nr} \end{array}$$

(2.2.2) $$\begin{array}{r} r_{it}=A_{2i}^{nr}cos\left(\theta_{t}-\theta_{pi}^{nr}\right)+C_{2i}^{nr} \end{array}$$

$A_{i}^{r},\theta_{pi}^{r}\;$and $C_{i}^{r}$ are parameters for rewarding trials (*r*), and $A_{i}^{nr},\theta_{pi}^{nr}$, and $C_{i}^{nr}$ are parameters for non-rewarding trials (nr). Equations (2.1.1) and (2.2.1) were fit for all units using Matlab function “regstats.” Units were considered significant if (2.1.1) and (2.2.1) differed (*p* < 0.05) from a constant model, that is if $A_{1i}^{r}$ or $B_{1i}^{r}\;$from Equation (2.1.1) and $A_{1i}^{nr}$ or $B_{1i}^{nr}$ from (2.2.1) were significantly different from zero (*p* < 0.05). Equation (2.1.2) was fit for all units using data from all rewarding trials during the transport stage (free 2D movement as the grasped object was moved to the target location). Similarly, Equation (2.2.2) was fit for all units using data from all non-rewarding trials during the transport stage. Both equations were fit using a nonlinear least squares fit. To minimize overfitting, data from the reaching stage were not included for analysis as all the reaching movements were the same, being made horizontally toward the right from the starting position. All estimated values and variances for parameters $A_{2i}^{r},\theta_{pi}^{r},C_{1i}^{r},\;A_{2i}^{nr},\;\theta_{pi}^{nr}\;$ and $C_{2i}^{nr}$ from (2.1.2) and (2.2.2) were calculated using non-linear least squares fit in Matlab. If any *A*_2*i*_ ($A_{2i}^{r}$ or $A_{2i}^{nr}$) was less than zero, then the value was multiplied by −1 to yield a positive value of *A*_2*i*_, and the corresponding θ_*pi*_was adjusted by:

$$\begin{array}{l} \theta_{pi,new}=\theta_{pi,old}+\pi \end{array}$$

After this adjustment θ_*pi*_ would always represent the preferred direction of the unit, meaning that the unit had a maximum firing rate when the direction of movement was θ_*pi*_.

For each unit, the shapes of the two tuning curves defined by Equations (2.1.2) and (2.2.2) could then be compared. *T*-tests were conducted to compare $A_{2i}^{r}$ with $A_{2i}^{nr}$ and $\theta_{pi}^{r}$ with $\theta_{pi}^{nr}$ for every unit to see if the amplitude or preferred direction were significantly different. For every unit that had a significant difference between either amplitudes or preferred directions between rewarding and non-rewarding trials, the difference between two preferred directions (Δθ_*pi*_) and the normalized difference between two amplitudes (Δ*A*_*i*_) were calculated:

$$\begin{array}{l} \Delta A_{i}=\frac{A_{2i}^{r}-A_{2i}^{nr}}{A_{2i}^{r}+A_{2i}^{nr}} \end{array}$$

Δθ_*pi*_ was the angle between two unit vectors $\left[cos\left(\theta_{pi}^{r}\right),sin\left(\theta_{pi}^{r}\right)\right]$ and $\left[cos\left(\theta_{pi}^{nr}\right),sin\left(\theta_{pi}^{nr}\right)\right]$:

(3) $$\begin{array}{r} \Delta \theta_{pi}=arccos\left(cos\left(\theta_{pi}^{r}\right)\cdot cos\left(\theta_{pi}^{nr}\right)+sin\left(\theta_{pi}^{r}\right)\cdot sin\left(\theta_{pi}^{nr}\right)\right) \end{array}$$

By this definition, Δθ_*pi*_ was in the range of [0, π].

For the manual task, during the grasping, transporting, and releasing scenes the linear force encoding model was given by:

(4) $$\begin{array}{r} r_{it}=\alpha_{i}f_{t}+b_{1i} \end{array}$$

where *r*_*it*_ is the *i*th unit's binned firing rate at time bin *t*, and *f*_*t*_ is the grip force at time bin *t*. Only *f*_*t*_ > 0 data were used for further analysis. Similar to the previous analysis, Equation (4) for all units was fit with the Matlab function “regstats” to find all significant units (*p* < 0.05). For every significant unit, the linear force encoding models for rewarding and non-rewarding trials were:

(4.1) $$r_{it}=\alpha_{i}^{r}f_{t}+b_{1i}^{r}$$

(4.2) $$r_{it}={\alpha_{i}^{nr}f}_{t}+b_{1i}^{nr}.$$

α_*ir*_ (rewarding) and α_*inr*_ (non-rewarding) for each unit were then compared using one-way analysis of covariance (ANCOVA, Matlab function “aoctool”) to see if the two slopes had a significant difference (*p* < 0.05). For each unit where the two slopes were significantly different, the normalized difference between the slopes Δα_*i*_ was calculated:

(4.3) $$\Delta \alpha_{i}=\frac{\alpha_{i}^{r}-\alpha_{i}^{nr}}{\left|\alpha_{i}^{r}|+|\alpha_{i}^{nr}\right|}.$$

#### Linear decoding model considering reward level

From these neural encoding models of rewarding and non-rewarding trials, a combined linear decoding/prediction kinematics model was designed taking multiple reward levels into consideration (decoder 2). For the velocity decoder, the decoding accuracies between decoder 1, where all trials were considered together, and decoder 2, treating rewarding and non-rewarding trials separately, were compared using 5-fold cross validation. For velocity decoder 1, the linear decoding model was:

(5) $$\begin{array}{r} \nu_{t}^{pre}=Mr_{t}+b_{2} \end{array}$$

For velocity decoder 2, the linear decoding models were:

(5.1) $$\nu_{t}^{pre}=M^{r}r_{t}+b_{2}^{r},\text{for rewarding trials and}$$

(5.2) $$\nu_{t}^{pre}=M^{nr}r_{t}+b_{2}^{nr},\text{fornon}-\text{rewardingtrials}.$$

$\nu_{t}^{pre}={\left[v_{xt}^{pre},v_{yt}^{pre}\right]}^{\prime }$ was the predicted velocity at time bin *t* and ***r***_*t*_ was the *n*-dimensional firing rate vector at time bin *t*, where *n* is the total number of units. For each testing data set, the velocity error at time bin *t* was:

$$\begin{array}{l} err_{vt}=\Delta v_{xt}^{2}+v_{yt}^{2},\\ \Delta v_{xt}^{2}={\left(v_{xt}^{pre}-v_{xt}\right)}^{2},\\ \Delta v_{yt}^{2}={\left(v_{yt}^{pre}-v_{yt}\right)}^{2}, \end{array}$$

where $v_{xt}^{pre}$ and $v_{yt}^{pre}$ were predicted velocities at time bin t and *v*_*xt*_ and *v*_*yt*_ were intended velocities at time bin *t*. For the single linear decoder (decoder 1), the total error was given by:

$$\begin{array}{l} err_{vT1}=\underset{T1}{\sum }err_{vt1}, \end{array}$$

where *T*_1_ was the total number of time bins for velocity decoder 1, which was the total number of time bins for all trials, and *err*_*vt*1_ was the velocity error at time bin *t* using decoder 1. For velocity decoders 2.1 and 2.2, we had:

$$\begin{array}{l} err_{vT2.1}=\underset{T2.1}{\sum }err_{vt2.1},\\ err_{vT2.2}=\underset{T2.2}{\sum }err_{vt2.2}, \end{array}$$

where *T*_2.1_ was the total number of time bins for velocity decoder 2.1 (all rewarding trials), *T*_2.2_ was the total number of time bins for velocity decoder 2.2 (all non-rewarding trials), *err*_*vt*2.1_ was the velocity error at time bin *t* using decoder 2.1, and *err*_*vt*2.2_ was the velocity error at time bin *t* using decoder 2.2. Since *T*_1_ = *T*_2.1_+*T*_2.2_, *p*_*ve*_ was defined to quantify the percent error reduction:

$$\begin{array}{l} p_{ve}=\left(1-\frac{err_{vT2.1}+err_{vT2.2}}{err_{vT1}}\right)*100. \end{array}$$

*p*_*ve*_ was used to compare the velocity decoding accuracy of decoder 1 and decoder 2.

The decoding accuracies between force decoders 1 and 2 were also compared using 5-fold cross validation.

For force decoder 1, the linear decoding model was:

$$\begin{array}{l} f_{t}^{pre}=H_{f}r_{t}+b_{3}, \end{array}$$

For force decoder 2, the linear decoding models were:

(6.1) $$f_{t}^{pre}=H^{r}r_{t}+b_{3}^{r},\text{for rewarding trials and}$$

(6.2) $$f_{t}^{pre}=H^{nr}r_{t}+b_{3}^{nr},\text{for non-rewarding trials}$$

$f_{t}^{pre}$was the decoded force at time bin *t* and **r**_*t*_ was the n-dimensional firing rate vector at time bin *t*, where *n* is the total number of units. For each testing data set, the force error at time bin *t* was:

$$\begin{array}{l} err_{ft}={\left(f_{t}^{pre}-f_{t}^{real}\right)}^{2}, \end{array}$$

where $f_{t}^{pre}$ was the decoded/predicted force at time bin *t* and $f_{t}^{real}$ was the real force at time bin *t*. The total errors for force decoders 1, 2.1 and 2.2 were given by:

$$\begin{array}{l} err_{fT1}=\underset{T1}{\sum }err_{ft1},\\ err_{fT2.1}=\underset{T2.2}{\sum }err_{ft2.1},\\ err_{fT2.2}=\underset{T2.2}{\sum }err_{ft2.2}, \end{array}$$

where *T*_1_ represented all time bins for force decoder 1 over all trials, *T*_2.1_ represented all time bins for force decoder 2.1 over all rewarding trials, *T*_2.2_ represented all time bins for force decoder 2.2 over all non-rewarding trials, *err*_*ft*1_ was the force error at time bin *t* using decoder 1, *err*_*ft*2.1_ was the force error at time bin *t* using decoder 2.1, and *err*_*ft*2.2_ was the force error at time bin *t* using decoder 2.2. Since *T*_1_ = *T*_2.1_+*T*_2.2_, *p*_*fe*_ was defined as:

$$\begin{array}{l} p_{fe}=\left(1-\frac{err_{fT2.1}+err_{fT2.2}}{err_{fT1}}\right)*100. \end{array}$$

Control groups were created to ensure that error reduction did not suffer because decoder 2 had more parameters than decoder 1. We shuffled the reward label for each trial randomly and then ran decoder 2 again. This way there were still two separate linear decoders, but the separation was random. *p*_*ve*_ and *p*_*fe*_ for the random shuffled decoder 2 were calculated using the same method as above and denoted by *p*_*ves*_ or *p*_*fes*_. This shuffling process was performed 1,000 times for each data block. Also, we compared the *p*_*ve*_ (for BMI task) and *p*_*fe*_ (for manual task) and the corresponding *p*_*ves*_ or *p*_*fes*_ distribution for each block using bootstrap hypothesis test. A *p*-value was computed from the percent of *p*_*ves*_ that are greater than *p*_*ve*_:

$$\begin{array}{l} p_{value}=\frac{n_{l}+1}{n_{t}+1}, \end{array}$$

where the total sample number *n*_*t*_ = 1000, and *n*_*l*_ is the number of samples whose *p*_*ves*_ were greater than *p*_*ve*_. Similarly, a *p*-value was computed from the percent of *p*_*fes*_ that were greater than *p*_*fe*_. This process was used to generate control groups for both the BMI and manual tasks.

#### Classification algorithm

Previous results showed that post-cue firing rates in M1 are separable between rewarding and non-rewarding trials (Marsh et al., 2015). A k-nearest neighbors (kNN) algorithm was used as a classifier, with firing rates from 0.3 to 0.9 s (6 time bins) after the cue as the input. The kNN algorithm was chosen because it is a nonparametric algorithm that allows for a nonlinear decision boundary, and also has an acceptable computational complexity for a small sample size such as the one used in this study, which has less than 200 samples (Altman, 1992). The time period from 0.3 to 0.9 s was chosen because for the manual task this was the time period when the virtual arm was horizontally moving to the right to reach the cylinder. For the BMI task, this was the time period when the virtual arm was moving horizontally to the right from the start position to the cylinder position. Since the virtual arm movement direction was always the same for that time period in the manual task and the cylinder position was always the same from the start position for the BMI task, the neural movement encoding was similar among trials.

For trial *t*, the input was the firing rate vector ***R***_*t*_. ***R***_*t*_ was the 6*n*-dimensional firing rate vector for trial *t*. Each element in ***R***_*t*_ represented one unit's firing rate for each of the six bins in the above mentioned time period. The output was the class label *l*_*t*_, where *l*_*t*_ = 0 for non-rewarding and *l*_*t*_ = 1 for rewarding trials. For any testing data ***R***_*test*_, the 5 nearest neighbors (Euclidean distance) of ***R***_*test*_ were found in training data and named as ***R***_***n*1**_ ~ ***R***_***n*5**_. We then obtained:

$$\begin{array}{l} l_{test}=\frac{1}{5}\overset{5}{\underset{i=1}{\sum }}l_{ni}, \end{array}$$

where *l*_*ni*_ were the labels for ***R***_***ni***_, *i* = 1, 2, 3, 4, 5 respectively. The test trial was classified as rewarding if *l*_*test*_ > 0.5, and classified as non-rewarding if *l*_*test*_ < 0.5.

Not all M1 units had reward modulation, so we hypothesized that a better classifier could be built by using the subset of units which showed reward modulation. To choose this optimal subset, the best individual unit ensemble construction procedure (Leavitt et al., 2017) was used. This consisted of: (1) building classifiers using each individual unit, (2) ranking all units based on the classification accuracy of that unit's classifier, and (3) iteratively adding ordered units to make a more complex classifier at each iteration. If the new unit added reduced the overall classification accuracy, it was dropped from consideration. The final classifier therefore used only the units that were beneficial to it, and did not use the remainder of the units.

#### Two-stage decoder

Combining the post-cue classifier (method 1.4.3) and the separate linear model (method 1.4.2), a two-stage decoder was designed to incorporate multiple reward levels. The first stage was the kNN classifier (method 1.4.3), using post-cue firing rates at the beginning of each trial to determine the reward level for the trial. The second stage then consisted of the two different linear decoders obtained from stage one. The second-stage decoder's output was velocity (using Equations 5.1 and 5.2) for the BMI task or force (using Equations 6.1 and 6.2) for the manual task. Using this two-stage decoder, the reward level could be calculated directly from the population firing rates, and no additional information was needed for the linear decoders in the second stage.

An offline test was run for the two-stage decoder, and its decoding accuracy was compared to a single stage linear decoder. *err*_*vt*_ and *err*_*ft*_ were calculated using the same equations as described previously. *p*_*ve*_ and *p*_*fe*_ for the two-stage decoder were defined as:

$$\begin{array}{l} p_{ve}=\left(1-\frac{\sum_{T}err_{vt3}}{\sum_{T}err_{vt1}}\right)*100,\text{and}\\ p_{fe}=\left(1-\frac{\underset{T}{\sum }err_{ft3}}{\underset{T}{\sum }err_{ft1}}\right)*100, \end{array}$$

where *T* represented the total number of time bins, *err*_*vt*3_ was the velocity prediction error at time bin *t* using the two-stage decoder, *err*_*vt*1_ was the velocity error at time bin *t* using the single linear decoder (velocity decoder 1), *err*_*ft*3_ was the force prediction error at time bin *t* using the two-stage decoder, and *err*_*ft*1_ was the force error at time bin *t* using the single linear decoder (force decoder 1). *p*_*ve*_ and *p*_*fe*_ were then used to test if this two-stage decoder had an improved accuracy over the single linear decoder. Table 1 depicts a flow charts of the three decoders.

**Table 1:**
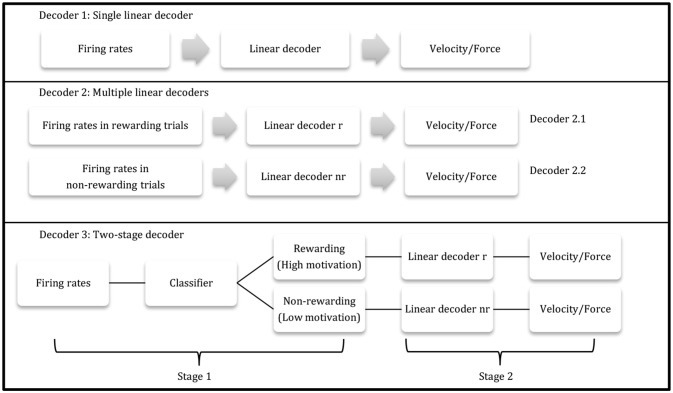
Three proposed decoders.

*Decoder 1 is a single linear decoder, decoding either velocity or force from M1 firing rates using a single linear decoder for all trials. Decoder 2 consists of two separate linear decoders, one for rewarding and one for non-rewarding trials. Decoder 3 is a two-stage decoder, which first classifies reward information from firing rate data then uses different decoders for different reward levels*.

## Results

The two NHP subjects in this work conducted two manual grip force sessions and two BMI sessions each. Only successfully completed trials were considered for analysis. For the manual grip force task, 64 M1 units were recorded in monkey P. There were 83 successful trials in session one with reward (R) [0,1] and 103 trials in session 2 with R [0,3]. In monkey S 77 M1 units were recorded. There were 152 successful trials for session one with R [0,1] and 130 trials in session two with R [0,3]. In addition, each NHP completed two BMI sessions. For monkey P, 102 M1 units were recorded over 64 successful trials in session one with R [0,1] and 72 trials in session 2 with R [0,3]. For monkey S, 87 M1 units were recorded over 66 successful trials for session one in BMI with R [0,1] and 145 trials in session two with R [0,3].

### Directional tuning curves change based on reward level under BMI control

**Figure 3:**
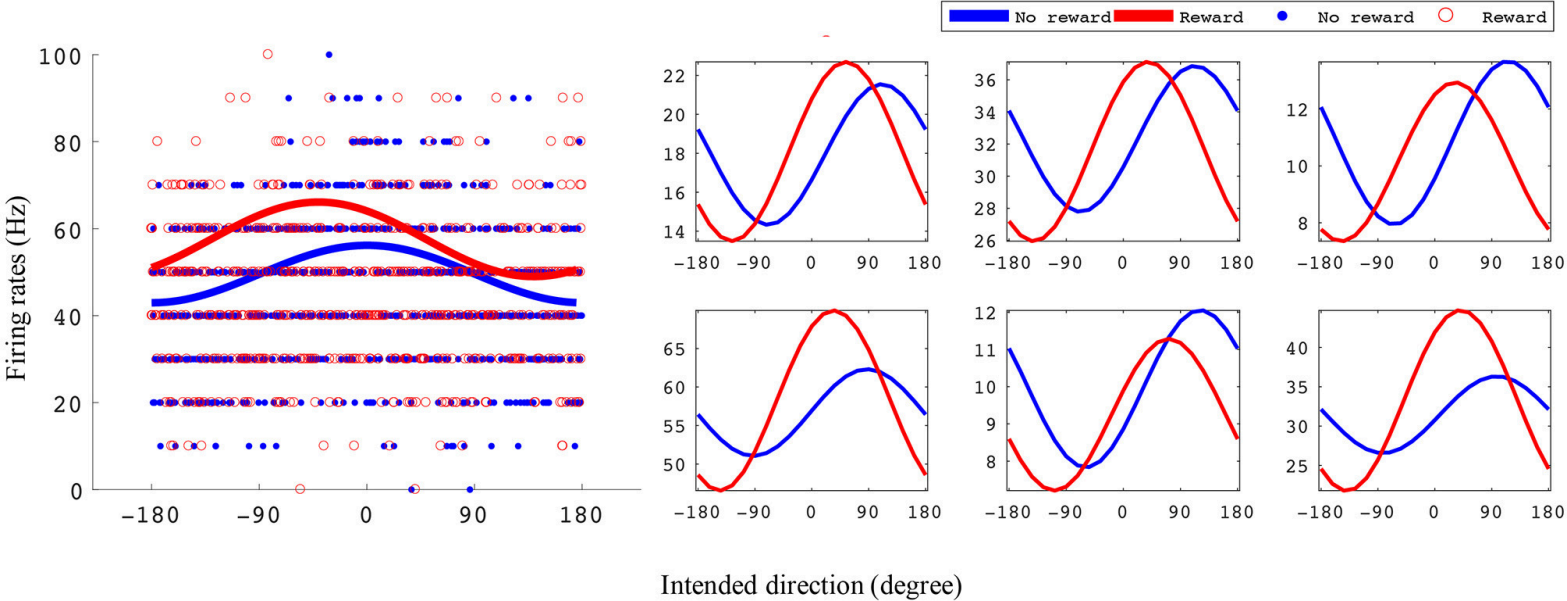
Example tuning curves for rewarding and non-rewarding trials for the BMI task. The x-axis is intended direction (degrees), and the y-axis is firing rate (Hz). The left subplots shows an example unit's tuning curves and all data points used to fit them. The right subplots are six example units' tuning curves. All example units were recorded from monkey S where the reward levels were 0 and 3.

**Figure 4:**
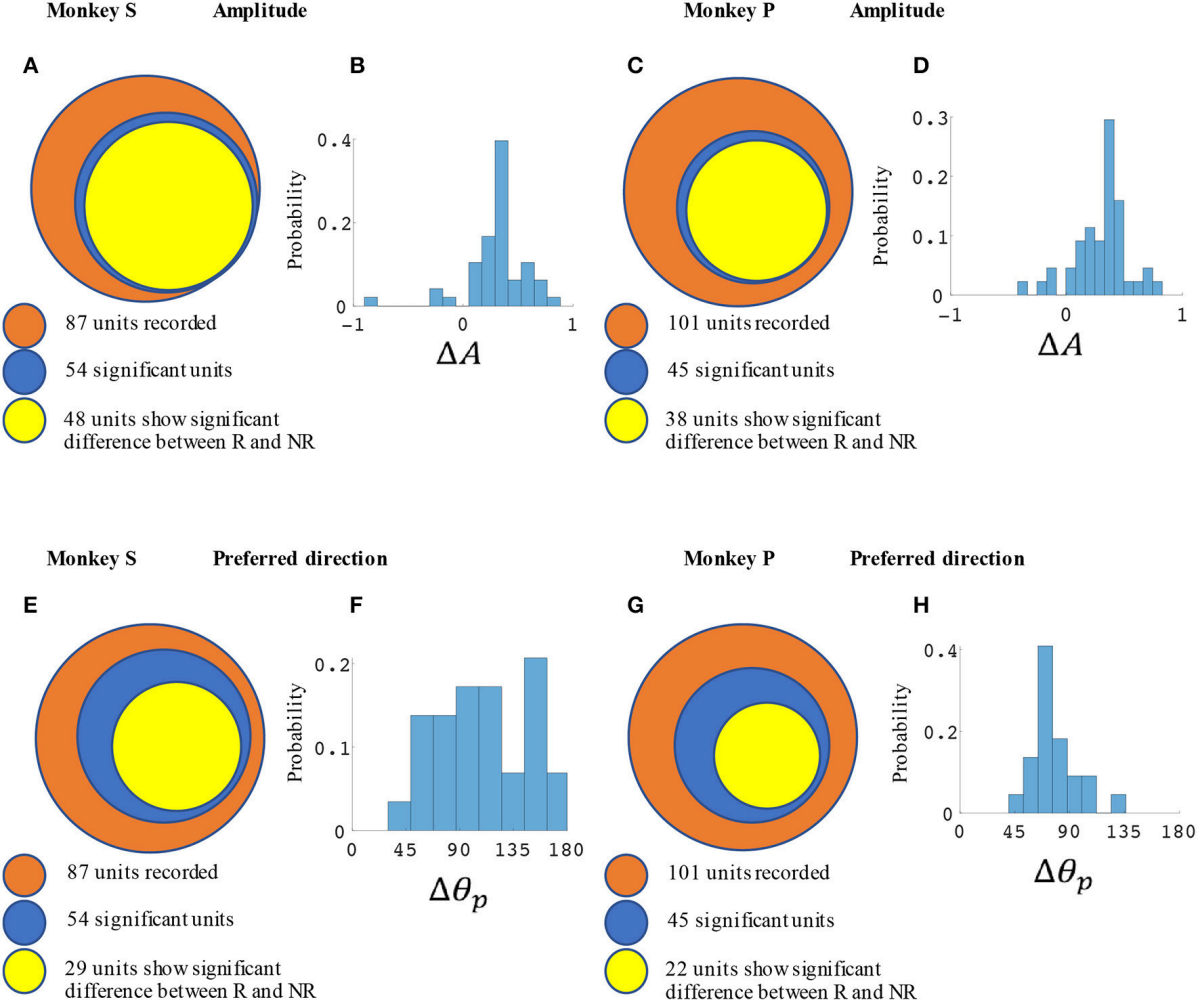
Statistical results for amplitude and preferred direction differences between rewarding (R) and non-rewarding (NR) trials in the BMI task. Monkey S **(A,B,E,F)** had a total of 52 significantly reward modulated units, and monkey P **(C,D,G,H)** had 42 units. **(A,C)** Show the number of units with significant changes in amplitude among all units and **(E,G)** show the preferred directions. **(B,D)** show the distribution of Δ*A* for all units with significant differences between R and NR. **(F,H)** show the distribution of Δθ_*p*_. All units were recorded in blocks where reward levels were either zero or three.

M1 units were used to decode movement information based on their tuning curves. Our hypothesis was that for a given unit, tuning curve parameters would change based on the presence or absence of cued reward. For the BMI task (Figure 2) a total of 87 units from monkey S and 102 units from monkey P were recorded. Of these, 54 units (62%) from monkey S and 45 units (44%) from monkey P had significant directional tuning (see Figures 3, 4). Of these significantly directionally tuned units, we then investigated if they showed significantly different tuning function parameters between the rewarding and non-rewarding trials. During the BMI task, a single BMI decoder was used. The following analysis refers to offline analysis of those BMI experiments. We found that monkey S had 29 units (33%) and monkey P had 22 units (22%) with significantly reward-modulated preferred directions under BMI control. A larger percentage of units had significant reward modulation of tuning curve model amplitude, with 48 units (55%) for monkey S and 38 units (37%) for monkey P. These results are summarized in Figure 4.

In Figure 3 we have plotted tuning curves for example units showing significant tuning functions that were also significantly reward modulated during the BMI task seen in Figure 2. For units with a significant difference between rewarding and non-rewarding trials in either amplitude or preferred direction, the distribution of the changes in amplitude (Δ*A*) and preferred direction (Δθ_*p*_) are shown in Figures 4B,F for monkey S and Figures 4D,H for monkey P. The distribution of Δ*A* indicates that, on average, tuning curve amplitudes are larger for rewarding trials than for non-rewarding trials.

### Reward level modifies force tuning curves

**Figure 5:**
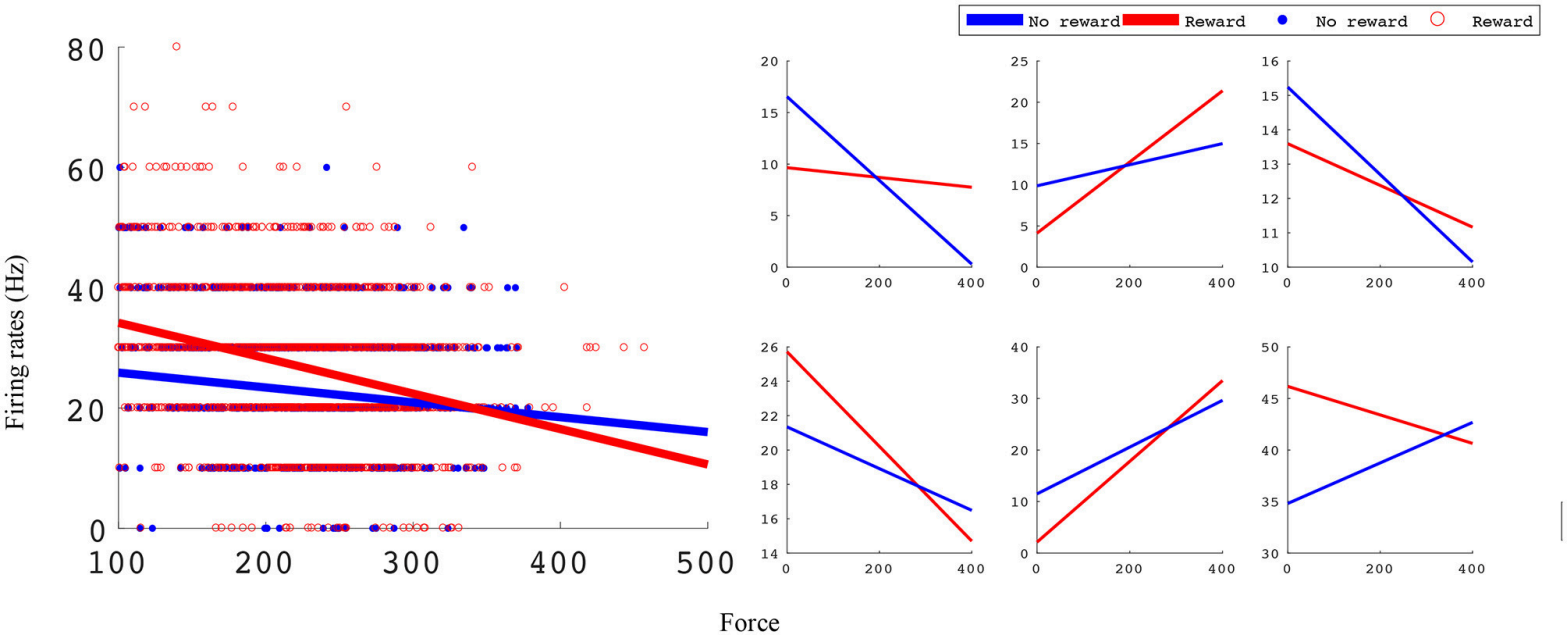
Linear force tuning curves between rewarding and non-rewarding trials for significant sample units. Both tuning curve characteristics, slope and intercept, change between rewarding and non-rewarding trials. The x-axis is the force sensor output, and the y-axis is firing rate (Hz). The left subplots shows an example unit's tuning curves and all data points used to fit them. The right subplots show six example units' tuning curves. All example units were recorded in monkey S M1 from an experimental block with reward levels of 0 and 3.

In the manual grip force task, M1 units encode force and value. Figure 5 shows linear force tuning curves for example units, fit using Equations (4.1) and (4.2).

**Figure 6:**
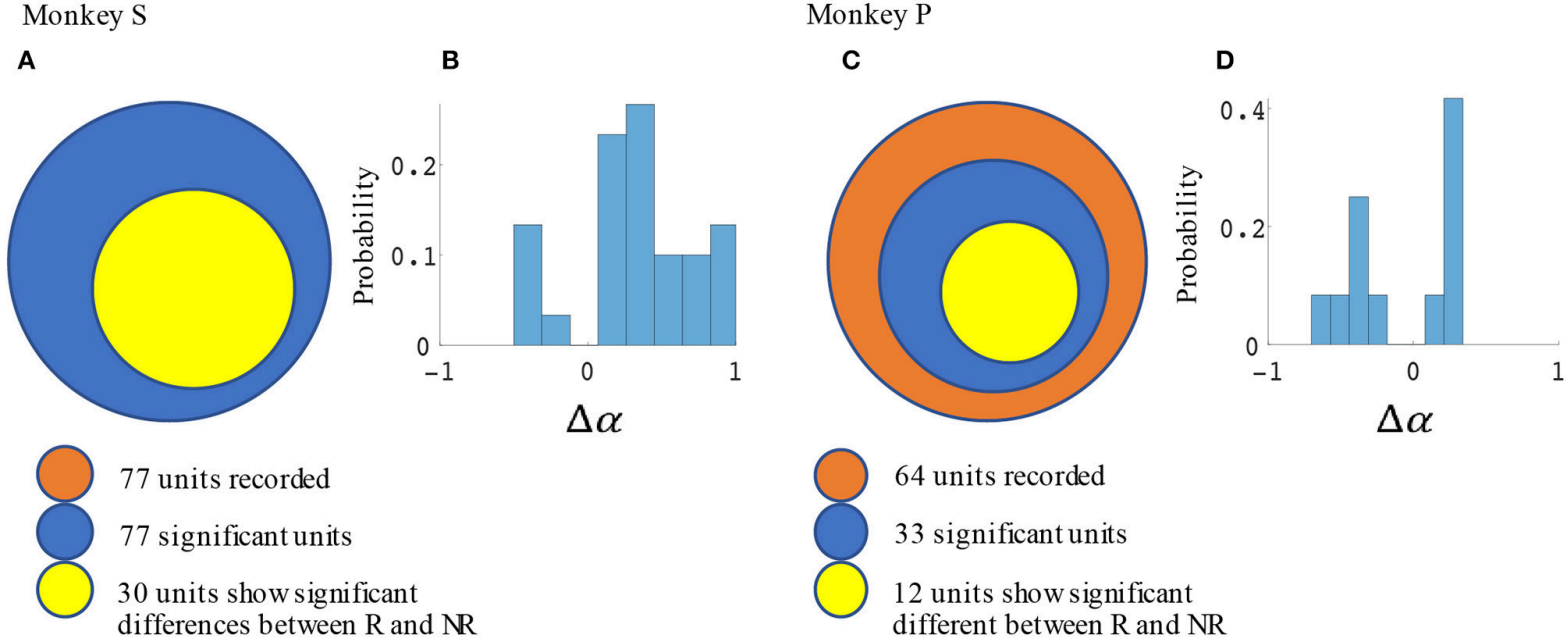
Statistical results for slope differences between rewarding and non-rewarding trials in the manual grip force task. The number of units with significant changes in force tuning curve slopes and Δα distribution are shown in **(A,B)** for monkey S, and in **(C,D)** for monkey P.

Significant force tuning was noted in 77 units (100%) in monkey S and 33 units (52%) in monkey P. Of these units with significant force turning, 30 units (39%) from monkey S and 12 units (19%) from monkey P were also significantly modulated by reward, having significantly different force turning curve slopes between rewarding and non-rewarding trials (see Methods section). Figure 6 summarizes these results and the distribution of changes in force tuning slopes Δα, which are the normalized differences between rewarding and non-rewarding slopes (see Equation 4.3).

### Incorporating reward level improves force and movement decoding accuracy

**Figure 7:**
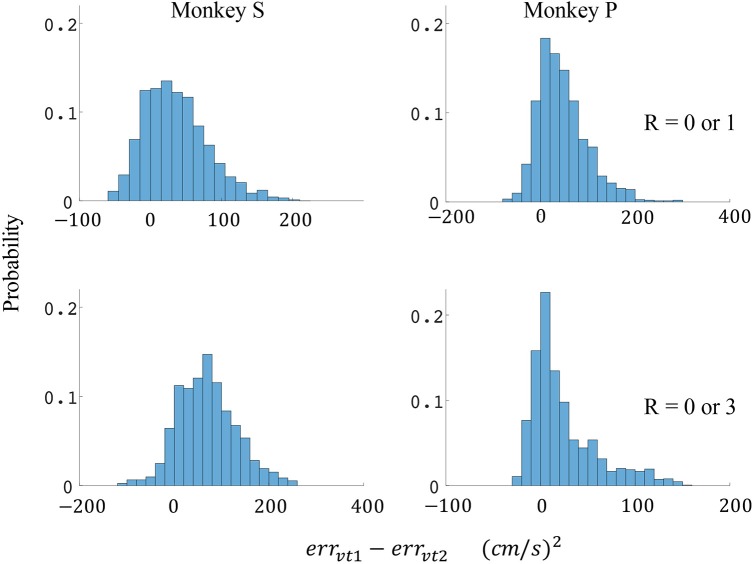
Distributions of velocity error reductions between decoder 1 and 2 (*err*_*vt*1_−*err*_*vt*2_). The x-axis represents velocity error reductions and the y-axis represents probability. The first row represents the task where the reward levels were 0 or 1. The second row represents the task where the reward levels were 0 or 3. The first column represents data from monkey S, and the second column represents data from monkey P.

**Figure 8:**
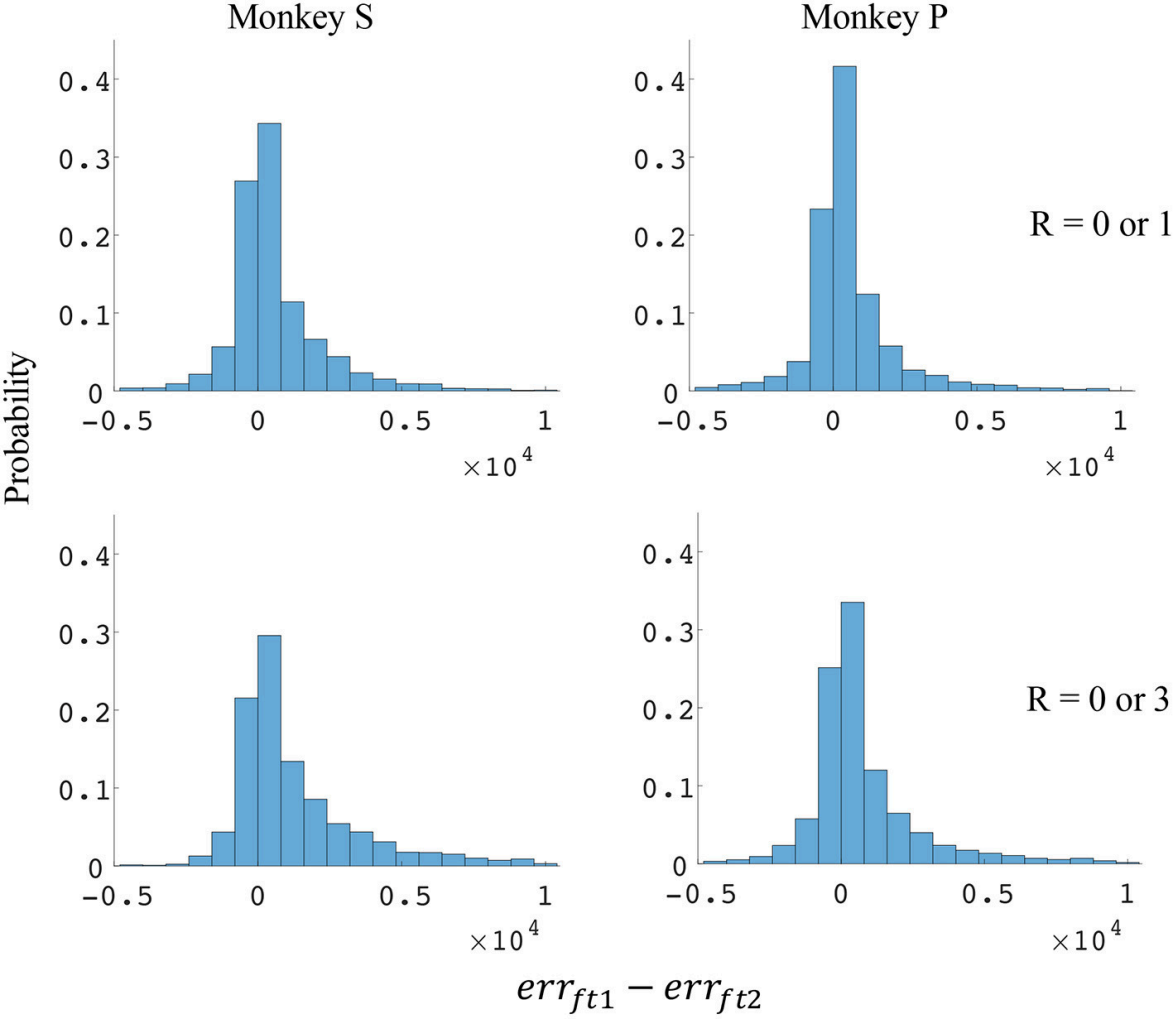
Distributions of force error reductions between decoder 1 and 2 (*err*_*ft*1_−*err*_*ft*2_). The x-axis represents force error reductions and the y-axis represents probability. The first row represents the task where the reward levels were 0 or 1. The second row represents the task where the reward levels were 0 or 3. The first column represents data from monkey S, and the second column represents data from monkey P.

The difference in directional tuning curves (Figures 3, 4) for individual units and the population suggest that units have different movement encoding for rewarding and non-rewarding trials, and that it may be helpful from a BMI decoding perspective to use models that allow for the influence of reward and motivation. Similarly, Figures 5, 6 show that units change their force tuning curves based on reward level. This suggests that if two separate linear decoders are used for velocity or force decoding, one for rewarding and one for non-rewarding trials, decoding accuracy will be improved compared to using a single linear encoder. This dichotomy was used to build a decoder that treated rewarding and non-rewarding trials separately (see Equations 5 and 6 in Methods section). Table 2 shows that decoding predictions were more accurate when we used the reward modulated decoders as compared to a single decoder (see Methods section). The percentage improvement in velocity decoding, *p*_*ve*_, and force decoding, *p*_*fe*_, was clear whether we used reward levels of zero and one, or zero and three (see Methods section). However, the percentage improvement was greater when the difference in trial value was greater, that is there was a greater percentage improvement between zero and three levels of reward than for zero and one level of reward. The reward modulated velocity decoder produced a 22–29% error reduction compared to the single linear decoder (Table 2, *p*_*ve*_). The reward modulated force decoder resulted in an error reduction of between 10 and 25% compared to the single force decoder (Table 2, *p*_*fe*_). These results demonstrate an improved decoding accuracy when rewarding and non-rewarding trials were treated separately, particularly when the value differences were greater. The distributions of decoding error differences between decoder 1 and 2 are plotted in Figure 7 (direction decoding, *err*_*vt*1_−*err*_*vt*2_) and Figure 8 (force decoding, *err*_*ft*1_−*err*_*ft*2_). These figures demonstrate that most of the data have positive error reduction using decoder 2 for both force and velocity decoding (*err*_*vt*1_−*err*_*vt*2_ > 0 or *err*_*ft*1_−*err*_*ft*2_ > 0). Additionally, we compared the error reduction between decoder 2 and shuffled surrogate data (see Methods section) where decoder 2 results are greater than that of the shuffled groups (*p*_*ve*_ or *p*_*fe*_ are larger than their corresponding *p*_*ves*_ or *p*_*fes*_), and this difference is significant (p < 0.05, bootstrap hypothesis test). These results obviate the need to accept the alternate explanation of improved decoding performance due to decoder 2 having more parameters than decoder 1.

**Table 2:** Decoding accuracy was greater when multiple linear decoders corresponding to different reward levels were used (decoder 2) compared to a single linear decoder (decoder 1), for both velocity and force decoding.

	**Monkey S (% error reduction)**	**Monkey P (% error reduction)**
*R* = 0 or 1, *p*_*ve*_	22	23
*R* = 0 or 1, $\bar{p_{ves}}$,	1.3	2.3
*R* = 0 or 3, *p*_*ve*_	29	27
*R* = 0 or 3, $\bar{p_{ves}}$	−1.1	0.29
*R* = 0 or 1, *p*_*fe*_	12	10
*R* = 0 or 1, $\bar{p_{fes}}$,	−0.35	1.3
*R* = 0 or 3, *p*_*fe*_	25	15
*R* = 0 or 3, $\bar{p_{fes}}$	−1.9	−2.2

*The control groups ($\bar{p_{ves}}$ or $\bar{p_{fes}}$) did not show any improvement*.

### Reward level is classifiable using post cue firing rates for cued BMI task

Results from Table 2 suggest that reward levels are useful information for BMI decoding. The classification accuracy and standard deviation across 10 Monte-Carlo repetitions for validation using post-cue firing rates to classify reward levels is shown in Table 3. For each Monte-Carlo repetition, 70% of the data were used for training and the remaining 30% for testing. All classification accuracies were greater than chance (50%). These results demonstrate that reward level can be classified using firing rates. A subset of units using the best individual unit procedure (Leavitt et al., 2017) provides more accurate results than when all units were used.

**Table 3:** Reward level classification mean accuracy and standard deviation across 10 Monte-Carlo repetitions using post-cue firing rates and classifying between rewarding (*R* > 0) and non-rewarding (*R* = 0) trials.

	**Using all units Mean accuracy ±s.d**.	**Using a subset of units Mean accuracy ±s.d**.
*R* = 0 or 1	70 ± 3.7%	73 ± 1.9%
*R* = 0 or 3	72 ± 2.8%	80 ± 2.4%

### Two-stage decoder can improve decoding accuracy in offline tests

**Figure 9:**
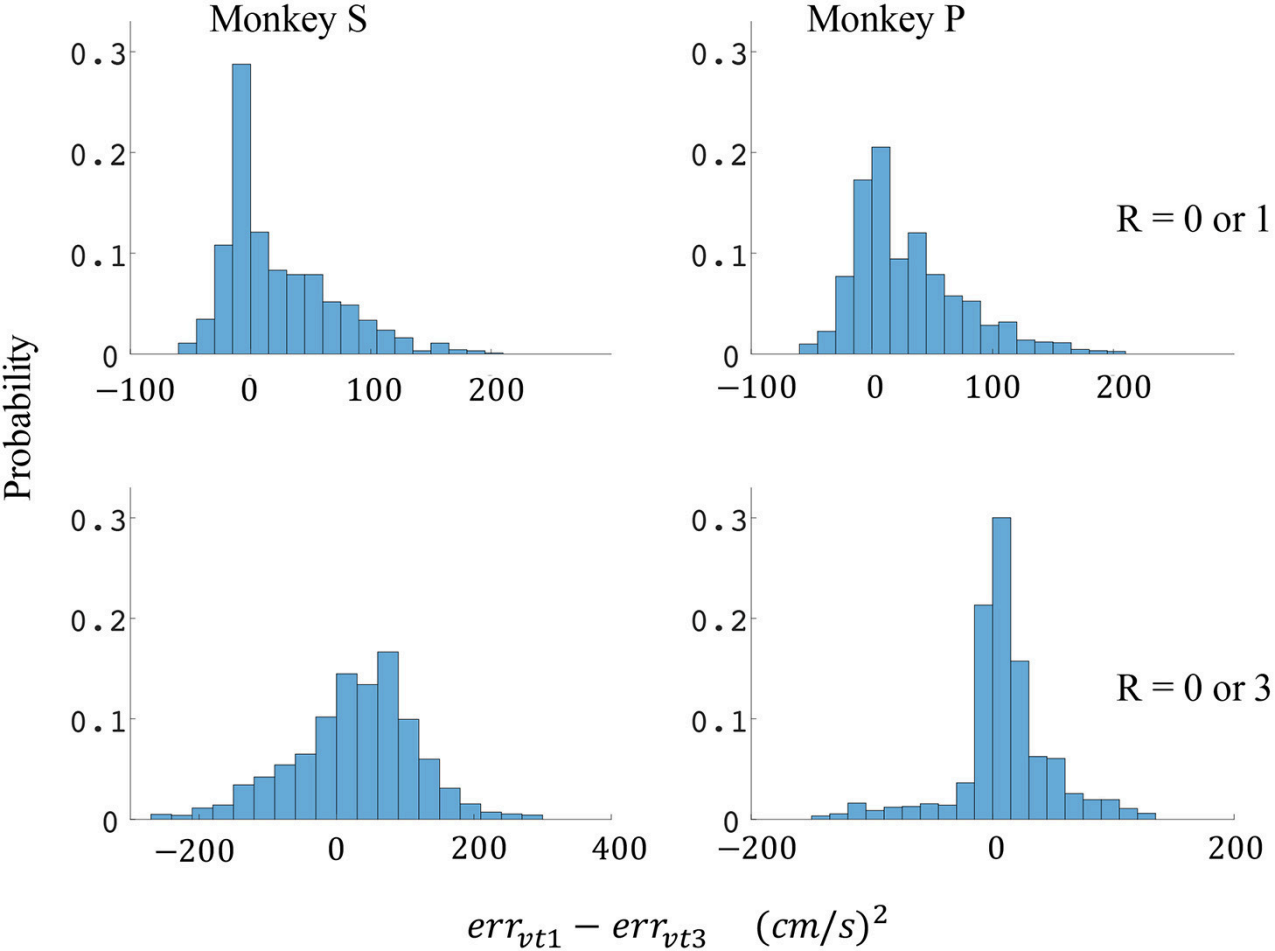
Distributions of velocity error reductions between decoder 1 and 3 (*err*_*vt*1_−*err*_*vt*3_). The x-axis represents velocity error reductions and the y-axis represents probability. The first row represents the task where the reward levels were 0 or 1. The second row represents the task where the reward levels were 0 or 3. The first column represents data from monkey S, and the second column represents data from monkey P.

**Figure 10:**
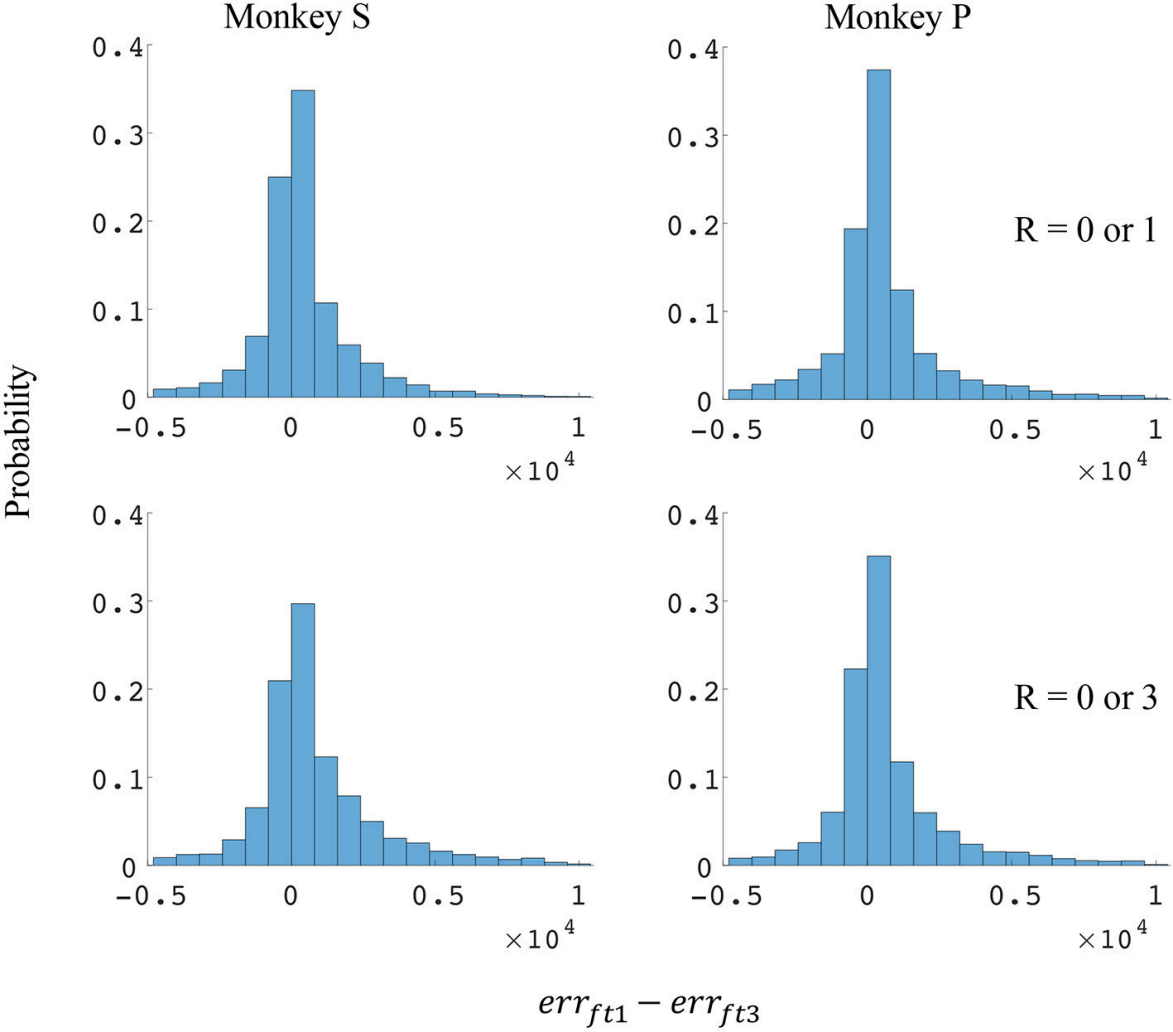
Distributions of force error reductions between decoder 1 and 3 (*err*_*ft*1_−*err*_*ft*3_). The x-axis represents force error reductions and the y-axis represents probability. The first row represents the task where the reward levels were 0 or 1. The second row represents the task where the reward levels were 0 or 3. The first column represents data from monkey S, and the second column represents data from monkey P.

Since the reward level could be classified using firing rates (Table 3), we developed a two-stage decoder. Table 4 shows velocity decoding improvement *p*_*ve*_ and force decoding improvement *p*_*fe*_ for the offline test of the two-stage decoder using 5-fold cross-validation for both monkeys. Here, the reward level is decoded during the first stage from the neural data and is used to determine the equations for the second stage. The percent improvements *p*_*ve*_ and *p*_*fe*_ are greater than 0 for all cases, therefore the two-stage decoder is more accurate than the single linear decoder (see Table 4). Plotted in Figures 9, 10 are histograms of the moment-to-moment decoding error difference between decoder 1 and 3 for direction decoding and force decoding respectively. Comparing with decoder 2 (Figures 7, 8) and decoder 3 (Figures 9, 10), we can see that more data has negative error reduction when using the two-stage decoder. This is because decoder 3 has a imperfect classifier, and misclassification can cause error increases (negative error reduction).

**Table 4:** Two-stage decoder improvement over decoder 1.

	**Monkey S (% error reduction)**	**Monkey P (% error reduction)**
*R* = 0 or 1, *p*_*ve*_	15	15
*R* = 0 or 3, *p*_*ve*_	7.9	7.2
*R* = 0 or 1, *p*_*fe*_	6.9	7.1
*R* = 0 or 3, *p*_*fe*_	10	7.3

*For both velocity and force decoding, the two-stage decoder reduced decoding error*.

## Discussion

In the current work, NHP subjects controlled either grip force manually or reaching kinematics with a BMI. In both cases the subjects controlled a simulation of an anthropomorphic robotic arm or hand to reach, grasp, and transport target objects. Each trial was cued as to the reward value the subject would receive for making the correct movement. In this work, we found that M1 unit activity was modulated by cued reward level in both the manual grip force task and the BMI kinematics control task. In both of these tasks the neural tuning functions, force tuning and kinematic tuning, were significantly modulated by the level of expected reward in blocks of trials where cued reward was (0 vs. 1), or (0 vs. 3). Our results indicate that reward influences motor related encoding in both manual and BMI tasks. When we explicitly took the influence of reward into consideration, our linear decoding models predicted with significantly higher accuracy. Having a more predicative linear decoding model is important because the most successful BMI systems use such linear models at their core, such as within a Kalman framework, or simply use the output of a linear model that decodes neural rate into movement parameters. A more accurate linear decoding model should lead to a more controllable BMI. In addition to the BMI control aspects of this work, the basic neuroscience is important, namely that expected reward modulates M1 motor related tuning functions, which was previously shown for kinematics in a manual task (Ramakrishnan et al., 2017). We show in the current work and in our previous work (Marsh et al., 2015; McNiel et al., 2016a,b; An et al., 2018) that the cued reward level in a task is classifiable using post-cue firing rates, therefore, it should be possible to build a classifier to determine the reward level before sending the neural activity through the appropriate BMI decoder. In one of these previous reports, we obtained 97% accuracy between rewarding and non-rewarding trial types by combining hybrid features made from local field potentials (LFPs) as well as single unit data (An et al., 2018). We have recently found that M1 activity in NHPs is also predictive of un-cued reward levels if these levels are predictable (Tarigoppula et al., 2018). This indicates that the system we are proposing, which uses a neural classifier to switch between BMI decoders, could have relevance past the laboratory setting with learned cues.

It can be seen from Table 3 that classification accuracy is higher when the difference between the reward levels is larger, that is between trials with cued reward of (0 vs. 3) as compared to trials with cued reward of (0 vs. 1). This indicates that neural firing rates have a greater degree of separability when there is a higher cued value. The same conclusion can also be inferred from Table 2, where both percent improvements for velocity decoding (*p*_*ve*_) and for grip force decoding (*p*_*fe*_) from trials with reward levels (0 vs. 3) are larger than (0 vs. 1). We have recently found that at least some M1 units code reward level in a linear manner in agreement with the above results (Tarigoppula et al., 2018). Table 2 shows decoding results for separate linear decoders where the reward level was specified by the experimenter as an additional input to the decoder. Table 4, on the other hand, shows decoding results for the two-stage decoder, where the reward level is automatically classified in the first stage using a kNN algorithm. The only difference between these two versions is whether the experimenter has provided additional information, or if the system did so autonomously. Since the reward level classifier for the two-stage decoder was not 100% accurate, the *p*_*ve*_ and *p*_*fe*_ from Table 4 are lower than the values in Table 2. As expected, the automatic classification of reward level comes at the cost of decreased decoding accuracy, indicating the value of a highly accurate classifier if this strategy is to be meaningful. We have previously shown that one can also obtain cued reward level information from local field potentials (Marsh et al., 2015; Tarigoppula et al., 2018) that are concurrently obtained when recording single units as discussed above (An et al., 2018), and we will incorporate such hybrid LFP/spike information in our online neural critic in future work.

From the current and previously cited work, it is clear that neural firing rates in M1 are modulated by more than just movement related information, and we have made use of reward information in a controlled environment to develop a more robust and accurate decoder. In this study, the virtual hand moved at a constant speed and animals could only control the movement direction in the BMI task. It is not possible that the firing rate differences are because of different movement speeds, but it could be related to intended movement speed. Previous work has already shown that there are reward induced changes in directional tuning under manual control (Ramakrishnan et al., 2017), and thus it is unlikely that intended speed alone would lead to the directional tuning differences observed under BMI control in the current work. Our results have shown that example units had different tuning curves between different reward levels. In other words, the units had different firing rates when the virtual hand was moving in the same direction and speed but when the reward levels were different. If we allowed monkeys to control the speed, their neural encoding models may have had a larger difference between different reward levels, and future work will test this. Intuitively, the intended speed will be larger for higher reward levels, just as movements and reaction times tend to be faster with higher reward (Watanabe et al., 2001). It is hard to say that the differences between neural encoding models shown in our study are only because of reward. It is possible that reward differences cause internal motor cortical changes, which then change neural representation. Regardless, we can capture these changes and build a better decoder by using reward levels as additional information. During BMI control, there were no extra visual stimuli as the reward based visual cues were displayed before BMI control started. Therefore, the unit-encoded differences due to reward level observed during BMI control are not likely due to visual stimuli.

In a more realistic BMI scenario the number of reward levels may not be known, and there may be other unknown factors encoded in M1. If reward levels are discrete, or can be treated as such, then it is possible to use a strategy similar to the two-stage decoder, but with multiple levels for reward using clustering as the first stage. If rewards are continuous, one possible solution is to use a latent variable model to represent reward value (Wu et al., 2009). It would be more meaningful to have a dynamic decoder that is able to filter out the reward based information for any level of reward and reject the effect of other such “non-movement” variables to obtain more purely movement relevant information for BMI control. We are currently working toward the goal of building a stable BMI decoder with these features that is able to function in a more complex, naturalistic environment.

## Ethics statement

All animal work was approved by the SUNY Downstate IACUC.

## Author contributions

YZ, JH, and JF conceived the research. YZ and JH conducted the experiments. YZ conducted the analysis. YZ, JH, JA, and JF wrote the paper.

### Conflict of interest statement

The authors declare that the research was conducted in the absence of any commercial or financial relationships that could be construed as a potential conflict of interest.
